# Case Report: Aspergillosis and *Cyathostoma americana* co-infection in the lower respiratory tract of a Harris’s Hawk (*Parabuteo unicinctus*)

**DOI:** 10.3389/fvets.2025.1680843

**Published:** 2025-10-29

**Authors:** Susanne Rau, Oliver Krone, Wieland Schrödl, Jan Schinköthe

**Affiliations:** 1 Faculty of Veterinary Medicine, Institute of Veterinary Pathology, Leipzig University, Leipzig, Germany; 2 Department of Wildlife Diseases, Leibniz Institute for Zoo and Wildlife Research, Berlin, Germany; 3 Faculty of Veterinary Medicine, Institute of Bacteriology and Mycology, Centre for Infectious Diseases, Leipzig University, Leipzig, Germany

**Keywords:** air sac, *Aspergillus fumigatus*, co-infection, dyspnea, helminths, pneumonia, raptors, trachea

## Abstract

A 12-year-old female Harris’s Hawk (*Parabuteo unicinctus*) that presented with prominent dyspnea and open-mouthed breathing with increasing respiratory problems during handling procedures died suddenly. Necropsy revealed extensive granulomatous airsacculitis, serositis, and pneumonia. Intralesional dark red helminths consistent with *Syngamidae* sp. were seen in the trachea, air sacs, lungs, and on top of multiple visceral organs. Furthermore, ascaridoid worms were found in the lumen of the small intestine. PCR analysis of both helminth species resulted in *Cyathostoma americana* and *Porrocaecum moraveci*, respectively. In addition, the air sacs contained innumerable areas of white to grayish sporulating mold. Mycological analysis revealed a prominent co-infection with *Aspergillus fumigatus*. While infection of the respiratory tract with *Cyathostoma americana* and air sac aspergillosis are known diseases in diverse raptor species, this case represents not only the first documented report of a Harris’s Hawk with pathological changes due to a prominent *Cyathostoma americana* infection but also of a raptor with Syngamidae-Aspergillus co-infection and concomitant endoparasitosis of the gut.

## 1 Introduction

Respiratory tract infections, caused by a wide range of pathogens, are frequently observed in avian species. Among these, infections due to *Aspergillus* sp. are the most common, with *Aspergillus fumigatus* being the most frequently isolated and pathogenic agent within this genus (1). Disease progression can vary from acute to chronic, depending on factors such as the number of inhaled spores, the bird’s age, and its immune status (2). Some bird species, including many birds of prey, appear to be more susceptible to Aspergillus infections than others (3). While acute aspergillosis typically results in rapid mortality, chronic infections may develop over several months and are characterized by granuloma formation in the lungs as well as thickened air sac membranes with plaques and nodules. Additionally, sporulation may occur in areas exposed to oxygen, leading to the visible formation of moldy lesions (4, 5). In raptors, clinical aspergillosis is frequently associated with stressors such as adverse husbandry conditions, intensive falconry training, malnutrition, or concurrent infections, and is a leading cause of mortality in captive birds (4). However, *Aspergillus fumigatus* can also act as a primary pathogen in birds without prior immune suppression or predisposing conditions (6, 7).

Nematode infestations of the respiratory tract, particularly involving syngamids, represent a well-documented phenomenon across a wide range of wild bird species globally. Among the genera within the Syngaminae subfamily, *Cyathostoma* is known to harbor the greatest number of described species (8). Although different *Cyathostoma* species have been reported in various parts of the respiratory tract of wild birds (9–13), it is *Cyathostoma americana* that is particularly associated with the trachea and air sacs of raptors (8, 14–17). Reports of the pathological significance of *Cyathostoma* spp. vary considerably, with some authors documenting subclinical infections characterized by low numbers of worms per individual (11, 12, 15, 16, 18, 19), while others describe strong inflammatory host reactions and even mortality resulting from a variable worm burden (10, 11, 16, 18). Although many authors suggest that, in addition to immunosuppression, bacterial and fungal co-infections are important risk factors for raptors developing fatal outcomes, only a few case reports actually support this claim by documenting such dual infections (13, 18). However, none of these reports describe clinically significant cases of co-infection with *Cyathostoma americana* and fungi associated with pathological alterations.

Unlike previous reports, this case highlights a rare and clinically significant co-infection with *Aspergillus fumigatus* and *Cyathostoma americana* in a raptor, associated with severe respiratory pathology. To our knowledge, this is the first documented instance of such a dual infection with histopathological evidence, suggesting a possible synergistic effect between fungal and parasitic agents in the avian respiratory tract.

## 2 Case description

### 2.1 Case presentation

A 12-year-old female Harris’s Hawk (*Parabuteo unicinctus*) from a private owner who kept approximately 10 different birds of prey was sent to our institution after its sudden death. Prior to this, the animal had presented with dyspnea and open-mouth breathing, both of which had increased significantly during handling procedures.

### 2.2 Necropsy

**Figure 1:**
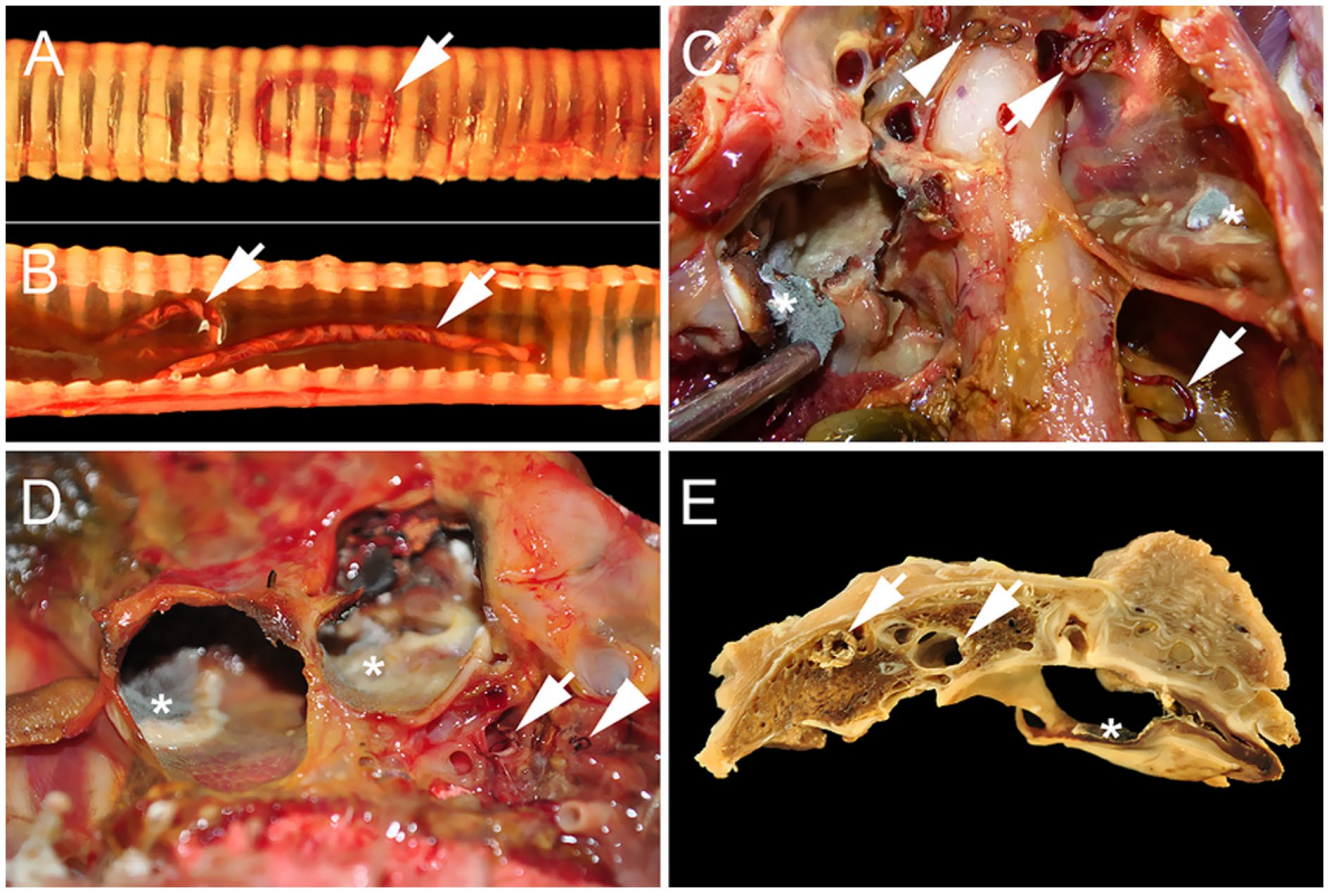
Syngamid and Aspergillus co-infection in the lower respiratory tract of a Harris’s Hawk (*Parabuteo unicinctus*) from Germany. **(A)** Adspection of the intact trachea shows a dark red worm within the tracheal lumen (arrow). **(B)** Two large dark red worms with an undulating white line resulting in a barber pole appearance (arrows) embedded in mucus lie within the lumen of the opened trachea. **(C)** Air sac membranes are brownish-black, up to 3 mm thick, and opaque with areas of white to gray fungal sporulation and formation of mycelia (asterisks). Lying freely within the lumen of the air sacs, there are numerous large, dark red female worms with an undulating white line resulting in a barber pole appearance (arrows) as well as one smaller, homogenously dark red male worm (arrowhead). **(D)** Opaque and brownish air sac membranes with areas of white to gray fungal sporulation and formation of mycelia (asterisks) with an adjacent female large dark red worm with an undulating white line resulting in a barber pole appearance (arrow) and two smaller homogenously dark red male worms (arrowhead). **(E)** Fixated transversal cut section of the body cavity at the level of the fourth rib. Consolidated lung tissue with big female worms inside the primary and secondary bronchi (arrows). Air sacs show prominent thickening and brownish discoloration as well as the formation of fungal mycelia (asterisk).

At necropsy, the bird showed a poor body condition indicated by severe muscular atrophy of the pectoralis muscle. Within the lower respiratory tract, more than 30 dark red strongyloids of two different sizes were seen. Five of the worms lay within the lumen of the trachea (Figures 1A,B), and a few others were embedded in the primary and secondary bronchi of the lungs (Figure 1D). However, for most of them, the worms were found lying freely in the air sacs (Figures 1C,D). Large worms measured up to 2.8 cm in length and featured a spiral staircase-like white line consistent with the reproductive tract against the dark red background of the rest of their body, delineating them as female worms (Figures 1C,D). Smaller worms, which were subsequently interpreted as male worms, were uniformly red and measured up to 1.0 cm in length (Figures 1C,D). Caudoventral lung parenchyma was consolidated and brownish-gray in contrast to craniodorsal areas, which were light pink and soft. Air sac membranes were thickened and colonized by fungal mycelia (Figures 1C–E). Similar lesions also involved the liver surface and liver sacs (Cava hepatica peritonei). Overall, alterations were consistent with prominent chronic granulomatous airsacculitis and serositis and mild pneumonia, with worm infestation and mycosis. In the gastrointestinal tract, six pale gray, round and pointed nematodes up to 6.5 cm long were present within the lumen of the small intestine without any visible concomitant inflammatory reaction.

### 2.3 Histopathology

Tissue samples collected during necropsy were fixed in 4% neutral-buffered formaldehyde and processed to formaldehyde-fixed and paraffin-embedded (FFPE) tissue blocks. FFPE blocks were cut at 3–4 μm to obtain tissue sections that were stained with hematoxylin and eosin. In addition, consecutive sections of lung and air sacs were stained by Grocott silver impregnation to visualize mycotic agents.

**Figure 2:**
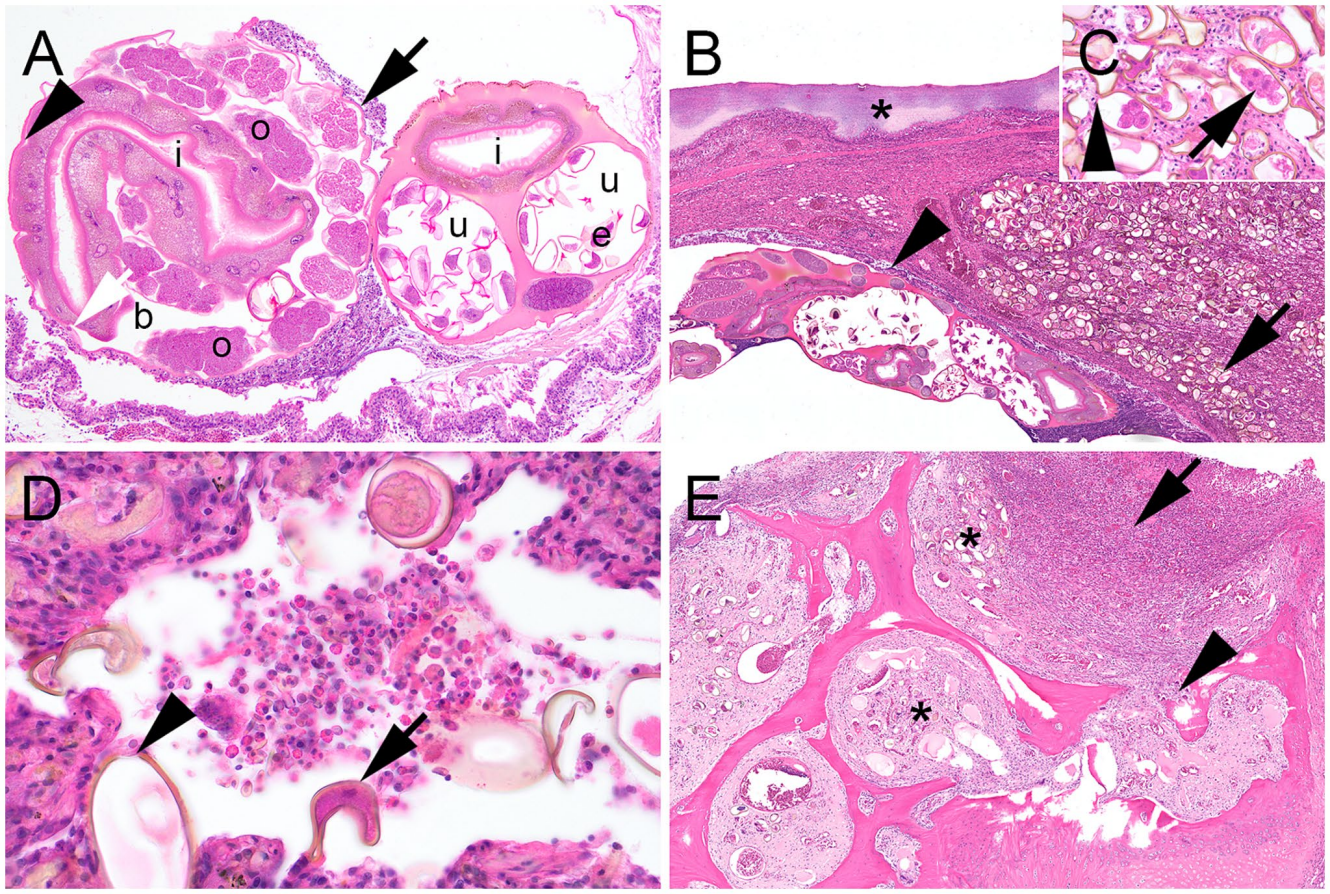
Histopathological lesions associated with a *Cyathostoma americana* infection in a Harris’s Hawk (*Parabuteo unicinctus*) from Germany. **(A)** Cross section of a female adult *Cyathostoma americana* in the trachea featuring an eosinophilic cuticula of 3 μm thickness (black arrow), a hypodermis with vacuolized lateral chords (white arrow), a platymyarian-meromyarian musculature (arrowhead), a body cavity (b), cut sections of ovaries (o) and uterus (u) with numerous developing thick-shelled eggs inside (e), a large intestinal tract (i) with a prominent oligocytous syncytous epithelium made out of few multinucleated vacuolized cells containing varying amounts of a light brown pigment, and a marked microvilli brush border. **(B)** Cut section of ventral areas of the lung with myriads of intraparenchymal thick-shelled operculated eggs measuring up to 80 μm × 40 μm and containing morulae within the parabronchi (arrow), female adult *Cyathostoma americana* in the lumen of the bronchus lined by respiratory epithelium (arrowhead), and adjacent prominently thickened air sac membrane (asterisk) with deposition of necrotic tissue, degenerated heterophilic granulocytes, and cellular debris. **(C)** Inset: Thick-shelled eggs with morula inside (arrow) and an operculum (arrowhead). **(D)** Lung with parasitic thick-shelled eggs (arrow) with opercula (arrowhead) and inflammatory cells within the lumen of parabronchi. Inflammatory cells are mostly composed of large, round cells with an eccentric nucleus and partially foamy cytoplasm, consistent with macrophages and heterophilic granulocytes, which are intermixed with cellular debris. **(E)** Body cavity with ribs and adjacent air sac. There is prominent osteomyelitis (arrow) with bone resorption (arrowhead), remodeling, and formation of woven bone. Parasitic eggs can be found within the medullary cavity (asterisks). **(A–E)**: Hematoxylin–eosin stain.

The overall histopathological findings concerning large strongyloid worms were consistent with those of adult female nematodes bearing the typical characteristics of true strongyles (Figure 2A). Apart from adult worms, myriads of intralesional thick-shelled operculated eggs were present within the trachea, the lung, and the air and liver sacs, but particularly within the parabronchi of ventral lung areas (Figures 2B,C). Surrounding the eggs and adult worms in the lungs, and also to a lesser extent in the trachea, there were high numbers of inflammatory cells aggregated with cellular debris (Figure 2D), as well as some foci of necrosis. These findings were consistent with the overall diagnosis of prominent granulomatous and necrotizing airsacculitis, serositis, and pneumonia. Additionally, the inflammatory response involved tissues adjacent to the lower respiratory tract, with infiltration of muscle and bone in the body wall and ribs (Figure 2E).

**Figure 3:**
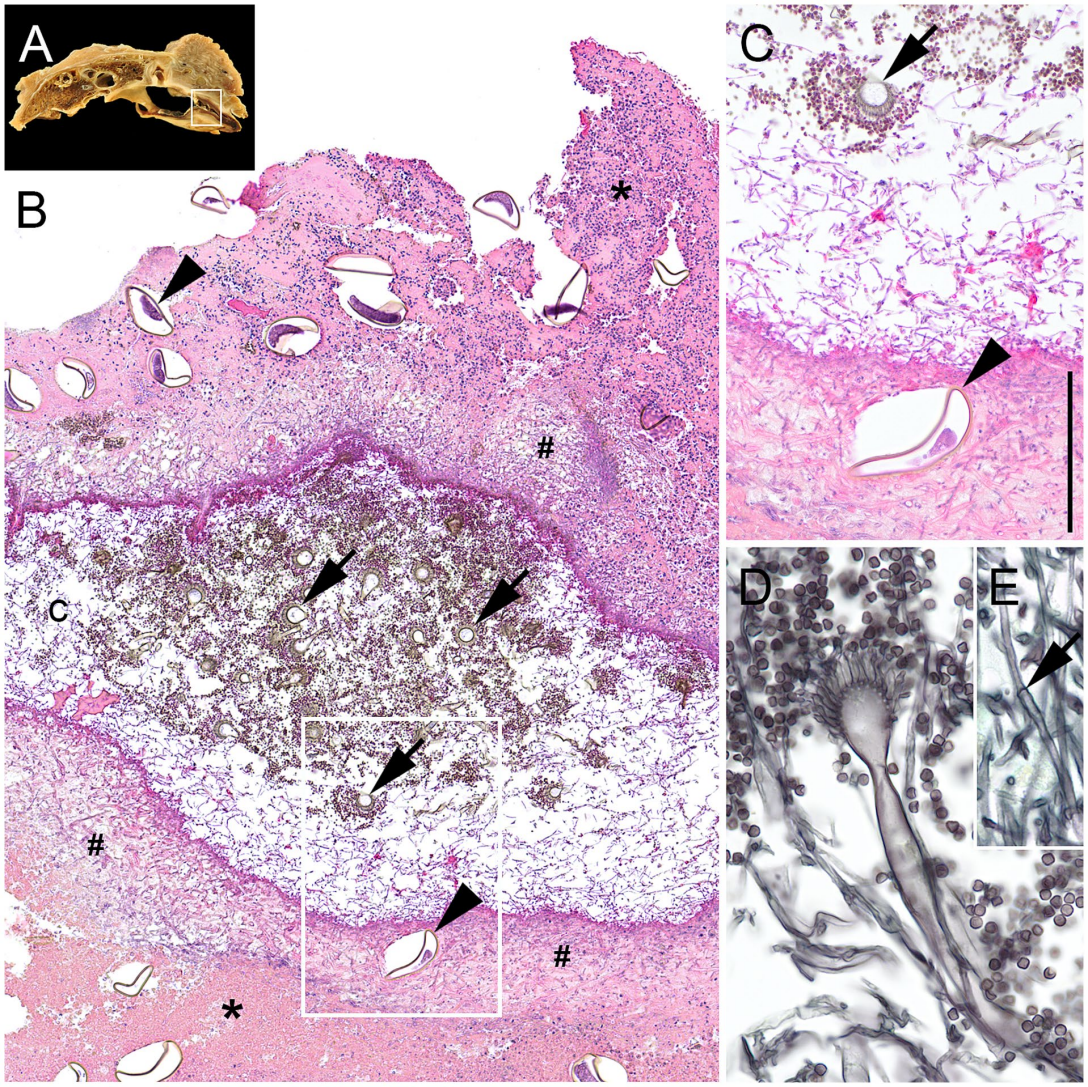
Histopathological lesions associated with an *Aspergillus fumigatus* infection in a Harris’s Hawk (*Parabuteo unicinctus*) from Germany. **(A)** Inset: Macroscopic image from 1E with airsacculitis (white rectangle). **(B)** The air sac membrane is up to 2,5 mm thick and extremely expanded by massive amounts of cellular debris, degenerated heterophilic granulocytes (asterisks) with myriads of embedded fungal hyphae (hash keys) as well as numerous thick-shelled parasitic eggs containing morulae (arrowheads), forming a central cavity (c), that is entirely filled with fungal hyphae as well as numerous conidial heads (arrows) and free conidia. **(C)** Higher magnification of the white rectangle in Figure 3B shows the central cavity filled with fungal hyphae, free conidia, and conidial heads (arrows) next to a parasitic egg with a morula (arrowhead) embedded in a thick layer (black line) of cellular debris, numerous fungal hyphae, and degenerated heterophilic granulocytes. **(D)** Detail of a typical sporulating *Aspergillus fumigatus* with a prominent conidial head and high numbers of surrounding free conidia. **(E)** Inset: Details of a typical *Aspergillus fumigatus* hypha with septation (arrow) and parallel walls. **(B,C)**: Hematoxylin–eosin stain. **(D,E)**: Grocott silver stain.

The air and liver sac membranes with mycotic mycelium were covered by massive amounts of necrotic debris, mostly consisting of degenerated heterophilic granulocytes, cellular debris, and necrotic tissue with embedded parasitic eggs, as well as varying amounts of fungal hyphae (Figures 2B, 3B,C). Hyphae were parallel-walled, septated (Figure 3E), measured up to 5 μm in thickness, and featured dichotome acute-angled branching. Additionally, abundant conidial heads and free conidia were present (Figures 3B–D). Both hyphae and conidia were argyrophilic (Figure 3D) and PAS-positive, thus histopathologically consistent with *Aspergillus* sp.

Specimens from the small intestine, where worms were found during dissection, were without any special findings. Worms themselves featured histological characteristics that classified them as adult ascarid nematodes.

### 2.4 Mycology

Representative samples from various macroscopically moldy areas within the air sacs were collected during post-mortem examination and initially stored at −20 °C. Collected specimens were then cultivated on blood agar and 2% Sabouraud dextrose agar. Cultivated material from all agar plates was examined multiple times microscopically and via MALDI-TOF-MS (MALDI Biotyper MBT smart, Bruker, Bremen, Germany). After 1 day, strong fungal growth was noted on Sabouraud agar plates. On day 2, strong fungal growth was present on all agar plates, including blood agar. Both multiple microscopical examinations and analysis via MALDI-TOF-MS unequivocally revealed *Aspergillus fumigatus* in all analyzed specimens.

### 2.5 Molecular analysis

Samples of both sizes of worms from the air sacs, as well as worm samples from the intestinal tract, were retrieved during post-mortem examination, stored at −20 °C, and later analyzed. Therefore, DNA from the sample material (~ 10 mg) was extracted using the DNeasy Blood & Tissue Kit (Qiagen, Hilden, Germany) according to the manufacturer’s protocol. Extracted DNA was stored at −20 °C until further processing. Fragments of ITS1-5.8S rDNA-ITS2 were amplified using primers (20) NLF (5‘-TTTGyACACACCGCCCGTCG-3′) and NLR (5‘-ATATGCTTAArTTCAGCGGGT-3′). Thermocycling conditions (21) were as follows: initial denaturation 95 °C/3 min; 48 °C/30 s, 72 °C/45 s—40 cycles; 72 °C/7 min—final extension. PCR was performed (25 μL) using 4 μL of genomic DNA, 12,5 μL of GoTaq^®^ [G2 HotStart (Promega, Madison, USA)], 0.5 μL of primer mix (NLF/NLR; 20 μM), and 8 μL of PCR H20. Purification of PCR products was performed using the NucleoSpin^®^ Gel and PCR clean-up kit (Macherey-Nagel, Düren, Germany), with DNA eluted in 25 μL of PCR H_2_O. Sequencing was performed in both directions (LGC Genomics, Teddington, UK), with consensus sequences extracted using BlastN. BlastN analysis of both samples produced 100% (bigger worm) and 99.10% (smaller worm) matches for *Cyathostoma americana* (Accession number: PV739826), respectively. The sample taken from the small intestine produced a 99.10% match for *Porrocaecum (P.) moraveci* (Accession number: PV739838).

## 3 Discussion

The avian respiratory tract is highly susceptible to infections due to several peculiarities of its anatomy, physiology, and immune system. Birds lack an epiglottis and a diaphragm, have reduced mucociliary clearance (22), and fewer luminal macrophages (23). Therefore, entering spores cannot be phagocytized and killed as efficiently as in mammals. Instead, birds rely on heterophils, which are less effective against fungi due to a lack of myeloperoxidase and catalase (24). Additionally, the high body temperature of birds in combination with the vast oxygenated spaces of the air sacs represents ideal conditions for fungal germination. The synergy of all these factors explains why infections with *Aspergillus* sp. rank among the most common pathologies of birds in general. Because spores of *Aspergillus fumigatus* are significantly smaller than those of other *Aspergillus* species, they are even more difficult to contain, thus making *Aspergillus fumigatus* by far the most frequent cause of such infections (1). While studies have shown that Aspergillosis rarely causes deaths (2%) in free-ranging raptors (25), infections with *Aspergillus fumigatus*, in contrast, represent the most common non-traumatic trigger of respiratory disease in captive raptors (5). This difference can be explained by multiple influencing parameters. While the most important risk factors for wild birds of prey—trauma due to collisions with human-made objects and emaciation (25)—are not as relevant to individuals held in captivity, the stationary character of their living situation brings numerous other problems with it. Especially poorly ventilated environments (2, 4) and intensive falconry training, transportation, and dietary changes (2, 26) represent important risk factors that facilitate respiratory disease. Furthermore, the etiological role of concomitant primary pathogenic agents is to be stressed particularly, as clinically affected animals oftentimes suffer from parasitical, viral, and/or bacterial co-infections (5, 6, 27), which was also true for the presented Harris’s Hawk, who suffered from a prominent nematode infection of the respiratory tract.

Infections with *Cyathostoma americana* occur through the ingestion of parasitic eggs, either taken up directly, e.g., from contaminated feces of passing free-living birds (16) or other infected captive birds housed in the same area, or indirectly, via the ingestion of paratenic hosts such as earthworms and arthropods. Some authors question whether the prey spectrum of raptors actually includes invertebrates and arthropods, suggesting instead that they ingest infectious developmental stages located within the intestines of their usual prey (19). Either way, adult worms consecutively live in the compartments of the respiratory tract, such as the trachea, air sacs, and lung, and produce eggs that are then coughed up and excreted with the feces (18). In the present case, all possible infection routes must be considered, as housing for falconry purposes usually allows contact with potentially contaminated soil and/or paratenic hosts, as well as with other possibly infected raptors housed nearby, and with free-ranging birds. Studies of the different endoparasitic species of wild raptors in Germany have shown that *Cyathostoma americana* (formerly called *Hovorkonema variegatum*) is found in up to 3% of wild raptor populations (11, 28). Outside Germany, few reports show infections of wild raptors with *Cyathostoma americana* in Canada (10, 18, 25), the USA and Czech Republic (21), Chile (15, 17) and the Netherlands (14), while evidence of infected captive birds is even more rare and recorded only in Canada (10) and Portugal (16). Of the few documented cases of birds of prey suffering from respiratory cyathostomosis, even fewer describe pathological changes and parasite burden. Overall, a wide spectrum of adult worm burden per individual is noted, ranging from mostly very few detected specimens (10, 11, 15, 18) up to over 180 worms per individual (10, 11, 15, 17, 18). All authors describe the air sacs as the predominant location for adult *Cyathostoma* infection, with fewer worms present within the trachea and the primary bronchi of the lung, and eggs being, in contrast, more numerous within parabronchi. The majority of these reports describe a (pyo-)granulomatous airsacculitis and pneumonia, even in cases with a relatively low worm burden, suggesting that this parasite is generally highly virulent (18). Interestingly, one report describes similarly low worm burdens with lymphoplasmacellular airsacculitis instead of prominent granuloma formation in various affected bird species (11), suggesting a rather low pathogenicity on the other hand. The morphological and histological findings in our case overall go in line with the observations made in the majority of the previously mentioned reports concerning the location and numbers of detected adult parasites and eggs, as well as the granulomatous character of the inflammatory reaction.

Interestingly, the worm burden and pathological changes in the present case differ significantly from those in the only other documented case of a *Cyathostoma* infection in the Harris’s Hawk species worldwide. In that report, which analyzed free-ranging birds from Chile, only one out of 29 examined Harris’s Hawks was found to be parasitized by seven adult *Cyathostoma americana* specimens (15). Although this relatively low worm burden primarily suggests a comparability to infections in other birds of prey that featured prominent associated granulomatous lesions at this infectious dose, the described bird surprisingly did not suffer from any pathological changes (15). A reason for this might be a lower susceptibility of the Harris’s Hawk species in general to *Cyathostoma americana* mono-infections. Alternatively, it cannot be excluded that the other birds of prey examined beforehand might have suffered from a concomitant, possibly immunosuppressing event which could not be detected at post-mortem examination, but might have facilitated the development of prominent pathological changes. Although such secondary bacterial, viral, fungal, or other parasitical co-infections are declared as an important reason to elicit prominent pathological changes in infected birds of prey by many authors (8, 16, 18), only very few published cases exist to actually underline this assertion, none of which describe a relevant fungal co-infection. Documented cases include clinically relevant bacterial co-infections (13, 17, 18). Fungal co-infections on the other hand are only mentioned by Lavoie (18) as an occasional association of *Aspergillus* sp. hyphae with pathological lesions in 1 out of 10 examined cases, and Fernando and Barta (19) who describe an association of fungal hyphae with cuticular remnants of parasites within parabronchi and secondary bronchi of birds infected with *Cyathostoma* sp., thus rendering the pathological contribution of the fungi to the actual demise of the birds questionable. This differs greatly from what we observed in our case, where innumerable intralesional hyphae as well as conidial heads and free conidia were present. Contrasting the described mono-infection with a low worm burden of 7 adult worms that did not cause any pathological changes in the Chilean Harris’s Hawk (15), we describe a significantly higher worm burden of 30 adult worms with a severe *Aspergillus fumigatus* co-infection, which most likely caused the poor body condition and muscular atrophy and led to the animal’s death. Unfortunately, we were unable to obtain information about the Harris’s Hawk’s housing conditions, which were not provided by the owner. Nevertheless, as aspergillosis is not a primarily contagious disease, we suggest that the presented infection with *Aspergillus fumigatus* occurred as a secondary event to a primary parasitic infestation of the respiratory tract with *Cyathostoma americana,* accompanied by immunosuppression and destruction of parenchymal integrity, both of which facilitated secondary fungal colonization.

Moreover, we want to highlight the *Porrocaecum moraveci* infection of the gastrointestinal tract, which adds another layer of complexity to this case. Within the genus *Porrocaecum,* phylogenetic analysis in recent reports reveals that more than 30 species can be differentiated. Some of these species, e.g., *P. moraveci*, *P. reticulatum*, or *P. angusticolle,* exist in certain countries of Europe, such as the Czech Republic (29, 30), and unspecified *Porrocaecum* sp. have also been reported in birds of prey in Germany (28). However, this represents the first identification of *P. moraveci* in a raptor from Germany, a new specimen of the *Porrocaecum* genus that was just recently described (30). While a mono-infection with worms belonging to the genus *Porrocaecum* is generally considered to be of low pathogenicity (29), we cannot rule out a disease-aggravating effect in the present case.

In this case report, we describe the first case of a clinically prominent *Cyathostoma americana* infection in a Harris’s Hawk worldwide. At the same time, we hereby present the first transcript of a concomitant significant fungal infection with *Aspergillus fumigatus* in a raptor species with a lethal outcome. A great strength of our multiparametric investigation is the identification and classification of worms by molecular methods, since most studies have relied on a few morphological criteria (10, 13–16, 18). Overall, this case highlights the relevance of nematode infestations with *Cyathostoma americana* within the respiratory tract of the Harris’s Hawk and underlines the importance of managing infections in captive raptors. The presence of both fungal and two nematode infections furthermore raises important questions about the potential for co-infections to exacerbate disease severity in raptors.

## Data Availability

The nucleotide sequences presented in the study are deposited in the NCBI GenBank repository, Accession number: PV739826, PV739838.
